# Ultrasound-guided lymphangiography and interventional embolization of chylous leaks following esophagectomy

**DOI:** 10.1515/iss-2018-0037

**Published:** 2019-03-09

**Authors:** Rolf Lambertz, De-hua Chang, Tilman Hickethier, Mahsa Bagheri, Jessica M. Leers, Christiane J. Bruns, Wolfgang Schröder

**Affiliations:** Department of General, Visceral and Cancer Surgery, University of Cologne, Cologne, Germany; Department of Radiology, University of Cologne, Cologne, Germany; Department of General, Visceral and Cancer Surgery, University of Cologne, Kerpener Str. 62, Cologne 50937, Germany

**Keywords:** chylothorax, esophagectomy, intranodal lymphangiography, Lipiodol, percutaneous embolization

## Abstract

**Objectives:**

Postoperative chylothorax is a serious complication after transthoracic esophagectomy, and is associated with major morbidity due to dehydration and malnutrition. For patients with high-output fistula, re-thoracotomy with ligation of the thoracic duct is the treatment of choice. Radiologic interventional management is an innovative procedure that has the potential to replace surgery in the treatment algorithm.

**Methods:**

Four patients with high-output chylous leaks following esophagectomy are presented. Ultrasound-guided lymphangiography with embolization of the thoracic duct and/or disruption of the cisterna chyli was performed to occlude the leakage site. Radiologic interventions and procedure-related outcomes are described in detail.

**Results:**

In all four patients, ultrasound-guided lymphangiography of the groin with injection of Lipiodol was able to detect and visualize the leakage site in the lower mediastinum. In three patients, the leak could be successfully occluded by Lipiodol embolization. In one patient, embolization failed and the disruption technique was successfully performed. No procedure-related complications were observed.

**Conclusions:**

In case of a postoperative chylothorax, radiologic intervention is feasible and safe. The procedure is indicated for high-output chylous fistulas after esophagectomy, and should be applied early after the diagnosis of this postoperative complication.

## Introduction

Transthoracic esophagectomy is generally accepted as a surgical standard treatment for patients with esophageal carcinoma. Despite improvements of surgical techniques, the procedure is still associated with a considerable rate of postoperative complications [1], [2]. Postoperative chylothorax is a rare event with a reported incidence of 1–9%; however, this complication has a severe impact on the general postoperative outcome due to a high rate of consecutive malnutrition and respiratory failure [3].

In case of a chylous fistula following transthoracic esophagectomy, the optimal management is still a matter of controversial discussion. According to the definition of the Esophagus Complication Consensus Group (ECCG), postoperative chylothorax can be classified as low- and high-output fistulas depending on the volume (< or>1000 mL/day) or as type I–III depending on the treatment [4]. In case of a low-output fistula, conservative management with parenteral nutrition and enteral application of medium-chained triglyceride diets are advocated (type I and II). For patients with high-output fistula, re-thoracotomy with ligation of the thoracic duct is recommended (type III). However, both therapeutic strategies are associated with certain disadvantages.

Recently, a novel technique has been described as the third treatment option for this postoperative complication, namely ultrasound (US)-guided lymphangiography with percutaneous Lipiodol embolization of the thoracic duct. Thus far, only very few publications have demonstrated the feasibility of this technique following esophagectomy, and its status with respect to the other two established treatment options remains unclear [5], [6], [7], [8], [9].

This report aims to analyze the postoperative outcome of four patients with chylothorax following esophagectomy, undergoing this novel radiological intervention in a high-volume center for esophageal surgery.

## Patients and methods

### Patients

Four patients with chylous leaks following esophagectomy were included in the retrospective analysis. The patients’ characteristics are displayed in Table 1. Three patients underwent transthoracic esophagectomy for esophageal carcinoma, and one patient had a transhiatal esophagectomy because of an esophageal perforation. The chylothorax was diagnosed between postoperative day (POD) 1 and 16. In all patients, the chylous leak was classified as high-output fistula.

**Table 1:** Patients’ characteristics and outcome.

	Patient 1	Patient 2	Patient 3	Patient 4
Age, years	44	76	47	54
Sex	M	M	M	M
ASA	III	III	III	III
BMI, kg bodyweight/m^2^	15.0	29.4	19.8	20.7
Surgical diagnosis	Perforation	AC	AC	SCC/AC
Multimodal treatment	No	No	Yes	Yes
Surgery	Transhiatal E	Hybrid IL E	IL E	McKeown E
Diagnosis of chylothorax, POD	1	7	5	16
Low-/high-output fistula	High	High	High	High
Maximum chylous effusion per day, mL	7000	5500	1000	3900
Postoperative morbidity (Dindo-Clavien)	IIIB	IIIA	IIIA	IIIA
Discharge, POD	120	28	55	56

M, male; ASA, American Society of Anesthesiologists; BMI, body mass index; IL, Ivor-Lewis; E, esophagectomy; POD, postoperative day; AC, adenocarcinoma; SCC, squamous cell carcinoma.

Due to anonymous data analysis, institutional review board approval and written informed consent were not required for this retrospective case study at the investigators’ institution.

### Radiologic technique of Lipiodol lymphangiography

**Figure 1:**
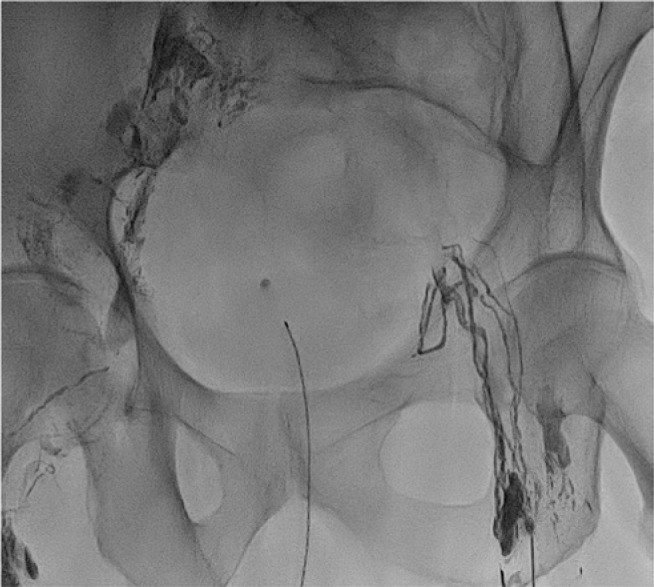
Bilateral groin lymphangiogram: after ultrasound-guided bilateral lymph node puncture, Lipiodol is infused into the lymphatics. The spot radiograph shows the initial phase with opacification of inguinal lymph nodes and lymphatic drainage of the pelvis.

**Figure 2:**
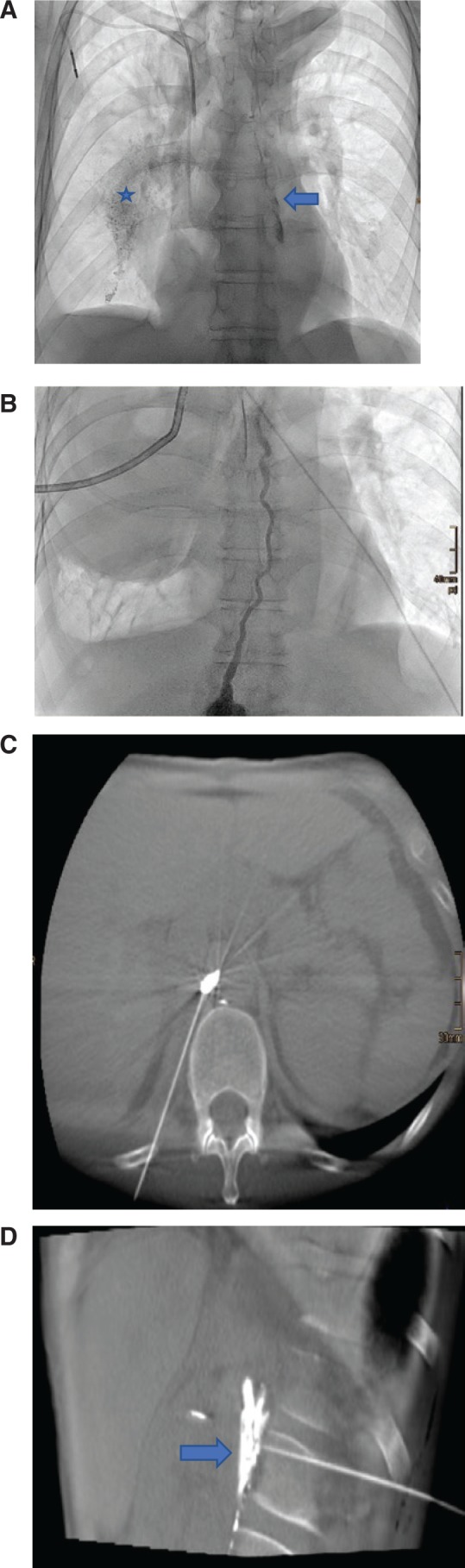
Radiologic intervention with detection and treatment of chylous leak. (A) Spot radiograph of the thorax showing leakage site at the mediastinum (arrow). Radiopaque Lipiodol accumulates in the pleural space on the right side (asterisk). (B) Spot radiograph showing the course of the thoracic duct in another patient. A right-sided chest drain was placed to evacuate a chylous pleural effusion. (C) Cone beam CT-guided puncture of the cisterna chyli. (D) Parasagittal multiplanar reformation shows the “needle disruption technique.” If the thoracic duct cannot be successfully cannulated for embolization after needle placement, multiple needle passes can be used to macerate the cisterna chyli. This promotes chyle flow into retroperitoneum, as shown by the extravasation (arrow), thereby allowing healing of the leak in the mediastinum.

In general, the thoracic lymphatic vessels were visualized through an injection of Lipiodol (Guerbet LLC, Bloomington, IN, USA) through an inguinal approach. The inguinal lymph node was identified using a high-frequency linear US transducer and punctured using a 20-G needle (Becton Dickinson S.A., Madrid, Spain) under US guidance. After verifying the position of the needle tip at the nodal hilum, Lipiodol was intermittently injected at a slow flow rate (approximately 2–3 mL/min) under fluoroscopy to a maximum of 10 mL (Figure 1). The time interval until the cisterna chyli and the thoracic duct became visible was about 20–30 min. Due to the fact that Lipiodol lymphangiography might also have a therapeutic effect and stop lymphatic leakage, the intervention was finished at this point. In case of a persistent leakage, a follow-up intervention using US-guided puncture of a lymph node at the contralateral groin and a repeat lymphangiography using Lipiodol was performed. In a second step, the thoracic lymphatic system was accessed through a percutaneous (cone beam) computed tomography (CT)-guided puncture of the contrast-filled cisterna chyli using a 21-G needle (Cook, Bloomington, IN, USA) during the same procedure or consecutively (Figure 2A–D). The attempt to advance a guide wire through the needle followed by a microcatheter into the thoracic duct failed. Therefore, embolization of the leak through the application of microcoils and/or glue was not possible. Instead, disruption/maceration of the cisterna chyli and/or the thoracic duct was used as an additional treatment option in one patient. For this purpose, the cisterna chyli and/or the thoracic duct were punctured several times using the above-mentioned needle to promote leakage of the high-viscosity, iodinated oil contrast agent, as well as to provoke a local hematoma in order to decrease the lymphatic flow and therefore allow the chest leak to occlude itself.

## Results

In all four patients, radiologic intervention was successfully carried out to treat the postoperative complication of a high-output chylous fistula following esophagectomy. None of the patients had complications associated with the radiological intervention. The details of the surgical and radiologic treatment are summarized in Table 2.

**Table 2:** Treatment of the chylous fistula.

	Patient 1	Patient 2	Patient 3	Patient 4
Initial treatment:	No	Yes	Yes	Yes
– Conservative management				
– Operative management	Thoracotomy, 2 POD; re-thoracotomy, 19 POD; laparotomy, 54 POD	No	No	No
– Radiological lymphangiography	87 POD	9 POD	48 POD	28 POD
– Unilateral (u) or bilateral (b) puncture of the groin	u	b	u	u
– Number (n) and size (mm) of injected nodes	1; 5	2; 10	1; 8	1; 10
– Identification of leak point, yes/no	Yes	Yes	Yes	Yes
– Max Lipiodol injection in ml	7	8	4	10
– Needle disruption technique, yes/no	No	No	Yes	No
– Embolization (coils/glue) of the thoracic duct, yes/no	No	No	No	No
– Total time of intervention, min	124	84	116	62
– Acute procedural complications (<24 h)	None	None	None	None
Treatment after first embolization:	700	200	800	4100
– Max chylous effusion/day, mL				
– Conservative/surgical management	Conservative	Conservative	Conservative	Conservative
– Repeated image-guided intervention (PID) via lymphangiography, yes/no	No	No	No	Yes (7)
Needle disruption technique, yes/no				No
Embolization (coils/glue) of the thoracic duct, yes/no				No
– Chest drain removal, POD	Right 29 POD	Right 7 POD, left 1 POD	Right 1 POD, left 5 POD	Right 11 POD, left 11 POD

POD, postoperative day; PID post interventional day.

Patient no. 1, a 44-year old man, was the only patient in whom surgical treatment of chylous leak preceded radiologic embolization. Following transhiatal esophagectomy for iatrogenic perforation of the distal esophagus, a high-output chylothorax was a diagnosed on POD 1. On POD 2, a right-sided thoracotomy was performed with ligation of the suspected thoracic duct in the inflamed lower mediastinum closely to the azygos vein. Due to a continuous chylous leak, re-thoracotomy was done on POD 19 without successfully closing the non-visible leak. After several weeks of conservative management with continuous leakage, the patient underwent laparotomy on POD 54 with ligation of the paraaortic lymphatic tissue at the level of the celiac trunk. Due to a persistent leak, radiological lymphangiography was done on POD 87. After US-guided puncture of a lymph node in the right groin and visualization of the thoracic duct with a leak in the lower mediastinum, 7 mL of Lipiodol was injected. On the following day, only 700 mL of pleural effusion was drained from the right thoracic cavity, and after complete termination of the leak with additional support of a fat-reduced diet, the thoracic drains were removed 7 days after the radiologic intervention. The patient was discharged on POD 120.

Patient no. 2, a 76-year old man, underwent a transthoracic hybrid Ivor-Lewis esophagectomy for an early adenocarcinoma of the distal esophagus. On POD 7, with the onset of postoperative enteral feeding with semi-solid food, a chylous fistula was diagnosed and conservative management of the chylothorax was started. Due to the high-output leak of >5000 mL/day and the reduced general condition, a decision was made to perform radiological intervention on POD 9. After bilateral puncture of the groin, a total of 8 mL Lipiodol was injected into two different lymph nodes. One day after the embolization, the chylous effusion stopped and the left and right chest drains could be removed on POD 9 and 16, respectively. After completion of the oral feeding program, the patient was discharged on POD 28.

Patient no. 3, a 47-year old man, was admitted from a rural hospital with the diagnosis of a chylothorax 43 days after hybrid Ivor-Lewis esophagectomy. Due to a locally advanced esophageal carcinoma, the patient had received neoadjuvant chemoradiation according to the CROSS protocol (41.4 Gy, paclitaxel/carboplatin). After several weeks of unsuccessful conservative treatment of high-output leak with approximately 1000 mL chylous fluid per day, the patient was scheduled for radiologic intervention, which was carried out on POD 48. After US-guided puncture of a lymph node in the right groin with subsequent injection of 4 mL Lipiodol, the leakage point could be clearly identified in the lower mediastinum. A CT-guided puncture of the cisterna chyli was performed aiming to occlude the thoracic duct. As it was not possible to catheterize the thoracic duct for selective embolization, the leak could be successfully closed by needle disruption of the cisterna chyli. Both thoracic drains could be removed during the following days, and the patient was discharged 7 days after the radiologic intervention.

In patient no. 4, a 54-year old man, a locally advanced adenocarcinoma of the distal esophagus and a squamous cell carcinoma of the proximal esophagus were diagnosed. After neoadjuvant chemoradiation, the patient underwent transthoracic esophagectomy with gastric reconstruction and cervical esophagogastrostomy (McKeown procedure). After an initially uneventful postoperative course, the patient developed a high-output chylous leak diagnosed on POD 16. After conservative treatment failed to stop chylous effusion, radiologic lymphangiography via the right groin was performed on POD 28. A total of 10 mL Lipiodol was injected after visualization of the leakage point in the lower mediastinum. However, during the following day, the leakage persisted with >4000 mL/day and image-guided intervention had to be repeated 7 days later. Again, 7 mL of Lipiodol was injected to finally close the leakage. Both right and left thoracic drains were removed 11 days after the second intervention, and the patient was discharged on POD 56.

## Discussion

### General management of chylothorax

Iatrogenic injury of the thoracic duct during cardiothoracic surgery is by far the predominant etiology of chylothorax. Chylous leaks after transthoracic esophagectomy are rare events; however, this postoperative complication is a life-threatening condition in particular for high-output fistula of >1000 mL/day and its reported 30-day mortality exceeds 17% [10]. Chylous fistula causes dehydration with electrolyte imbalance, malnutrition, as well as lymphopenia with an impaired immunological status and consecutive pneumonia. Owing to the low incidence of this complication, no generally accepted treatment algorithm has been established thus far. Conservative management with reduction of the enteral loading including a fat-reduced diet and adequate parenteral fluid administration is considered as standard care for low-output (type A) leaks according to the ECCG classification [4]. The medical management of high-output (type B) leaks is difficult because of the severe fluid loss; thus, surgical occlusion of the leaking thoracic duct is necessary. However, due to anatomical variations or the concomitant mediastinitis with inflammatory tissue, the leakage site cannot always be detected intraoperatively. In addition, as the surgical procedure of a re-thoracotomy itself is associated with considerable morbidity, the timing of surgery is still a matter of discussion.

### Technique of interventional lymphangiography and embolization

In this setting, radiologic intervention seems to be a favorable option completing the present treatment strategy. Clinical experience with intranodal lymphangiography followed by embolization of the thoracic duct or disruption of the cisterna chyli is limited, and only a small number of case series have been published thus far. Lymphangiography aims not only to localize the chylous fistula but also offers the possibility to embolize and thereby cure the chylous leak. The interventional technique is based on the visualization of the thoracic lymphatic vessels through an injection of Lipiodol via both a pedal and an inguinal approach [5], [6], [11]. The latter offers a more feasible access to the lymphatic system, reduces the radiation dosage due to the shorter time of investigation, and therefore has replaced pedal lymphangiography nowadays [12]. Although the use of significantly higher amounts of Lipiodol of up to 40 mL has been reported, in this series of four patients, the maximum of 10 mL Lipiodol was sufficient to localize the leakage site [13]. After injection of Lipiodol, an observation period of 2–5 days is justified because Lipiodol application itself has a therapeutic effect and might occlude the lymphatic leakage [14]. A second option to occlude the visualized leak is the percutaneous (cone beam) CT-guided puncture of the contrast-filled cisterna chyli with catheterization of the thoracic duct and mechanical obstruction of the leakage site by microcoils and/or a mixture of iodized oil and N-butylcyanoacrylate [15], [16]. In the largest series published by Itkin et al., the technical success rate of thoracic duct catheterization was 67%, and the clinical success rate of thoracic duct embolization by either technique to occlude the chylous leak was as high as 90% [5]. In case of a persisting chylothorax, disruption/maceration of the cisterna chyli and/or the thoracic duct has been described as an additional treatment option [17]. The possible complications of the described intervention include the development of an arterio-venolymphatic shunt with consecutive Lipiodol embolism as well as bleeding from the puncture sites. None of these complications were observed in the four patients of this series.

### Treatment algorithm including radiologic embolization

The present series of four cases demonstrates the feasibility and safety of this interventional technique and confirms previous reports [6], [8], [9]. However, despite these encouraging results, the limitations of case reports must be acknowledged. It is important to realize that US-guided lymphangiography with thoracic duct embolization requires an experienced interventional radiologist to perform this complex and time-consuming procedure. With increasing centralization of esophageal cancer surgery, interventional radiologists with expertise in this field need to become part of the team approach for this tumor entity, and a close cooperation of these two specialties is important to decide on the best treatment option. Although it is difficult to compare the success rates of the surgical and radiologic approach due to the low incidence of this postoperative complication, interventional thoracic duct embolization should precede the surgical approach in patients with a high-output fistula. The rationale of this strategy is the less invasive character of this intervention and therefore lower complication rate compared to re-thoracotomy or re-thoracoscopy. For patients with low-output fistulas of only a few hundred milliliters per day, the conservative approach with standardized dietary pathways is still the preferred strategy.

## Conclusion

US-guided lymphangiography with opacification of the retroperitoneal lymphatic system for subsequent thoracic duct embolization is feasible, reproducible, and less invasive compared to any proposed surgical treatment of a postoperative chylous fistula, and therefore should be considered as the first-line treatment of this serious complication following esophagectomy.

## Supplementary Material

The article (iss-2018-0037) offers reviewer assessments as supplementary material.
