# Supplementary material for: Ultrasound-guided lymphangiography and interventional embolization of chylous leaks following esophagectomy
Source: Innov Surg Sci. 2019 Mar 9;4(3):85–90. doi: 10.1515/iss-2018-0037 (PMC6817730; doi:10.1515/iss-2018-0037)
Supplement: Supplementary file 1 [file iss-4-20180037-s001.pdf]

Reviewer Assessment

Rolf Lambertz, De-hua Chang, Tilman Hickethier, Mahsa Bagheri, Jessica M. Leers, Christiane J. Bruns and Wolfgang Schröder*

Ultrasound-guided lymphangiography and interventional embolization of chylous leaks following esophagectomy

https://doi.org/10.1515/iss-2018-0037
Received November 8, 2018; accepted January 22, 2019

***Corresponding author: Wolfgang Schröder,** Department of General, Visceral and Cancer Surgery, University of Cologne, Kerpener Str. 62, Cologne 50937, Germany, E-mail: wolfgang.schroeder@uk-koeln.de

Reviewers' Comments to Original Submission

Reviewer 1: Timm Franzke

Nov 12, 2018

**Reviewer Recommendation Term:**	Accept
**Overall Reviewer Manuscript Rating:**	N/A
**Custom Review Questions**	**Response**
Is the subject area appropriate for you?	5 - High/Yes
Does the title clearly reflect the paper's content?	5 - High/Yes
Does the abstract clearly reflect the paper's content?	5 - High/Yes
Do the keywords clearly reflect the paper's content?	5 - High/Yes
Does the introduction present the problem clearly?	4
Are the results/conclusions justified?	5 - High/Yes
How comprehensive and up-to-date is the subject matter presented?	4
How adequate is the data presentation?	3
Are units and terminology used correctly?	5 - High/Yes
Is the number of cases adequate?	1 - Low/No
Are the experimental methods/clinical studies adequate?	4
Is the length appropriate in relation to the content?	4
Does the reader get new insights from the article?	5 - High/Yes
Please rate the practical significance.	4
Please rate the accuracy of methods.	3
Please rate the statistical evaluation and quality control.	4
Please rate the appropriateness of the figures and tables.	4
Please rate the appropriateness of the references.	4
Please evaluate the writing style and use of language.	4
Please judge the overall scientific quality of the manuscript.	4
Are you willing to review the revision of this manuscript?	Yes

Comments to Authors:
Thanks for this article.

Open Access. © 2019 Lambertz R., et al., published by De Gruyter. CC BY This work is licensed under the Creative Commons Attribution 4.0 Public License.

Reviewer 2: Andrea Beuleke

Jan 18, 2019

**Reviewer Recommendation Term:**	Reject
**Overall Reviewer Manuscript Rating:**	20

**Custom Review Questions**	**Response**
Is the subject area appropriate for you?	4
Does the title clearly reflect the paper's content?	5 - High/Yes
Does the abstract clearly reflect the paper's content?	4
Do the keywords clearly reflect the paper's content?	4
Does the introduction present the problem clearly?	3
Are the results/conclusions justified?	3
How comprehensive and up-to-date is the subject matter presented?	4
How adequate is the data presentation?	3
Are units and terminology used correctly?	4
Is the number of cases adequate?	1 - Low/No
Are the experimental methods/clinical studies adequate?	3
Is the length appropriate in relation to the content?	4
Does the reader get new insights from the article?	4
Please rate the practical significance.	4
Please rate the accuracy of methods.	N/A
Please rate the statistical evaluation and quality control.	1 - Low/No
Please rate the appropriateness of the figures and tables.	4
Please rate the appropriateness of the references.	3
Please evaluate the writing style and use of language.	4
Please judge the overall scientific quality of the manuscript.	3
Are you willing to review the revision of this manuscript?	No: time management

Comments to Authors:

This article describes an exciting technique to treat postoperative high-output chylous fistulas which is far less invasive than a re-thoracotomy. Therefore it has a clearly reduced morbidity and mortality while having a successrate as high as 90%. On the other hand it is a time consuming procedure and needs an highly specialized radiologist. Despite it being a small report of only 4 patients the authors conclude that the embolization with Lipiodol should be the first line therapy in postoperative chylous leaks. Although the cases presented show a 75% success of embolization and in one case of mazeration of the cisterna chyli further studies with a higher number of cases will be needed.

Minor Comments:

Use of different classifications describing the type of chylous leakage (type I-III , later - in the discussion - described as Type A/B). Would it be better to stay in one classification?

In one of the four cases the authors used an alternative technique (Mazeration), this should be mentioned in the introduction.
